# Clinical *S. aureus* Isolates Vary in Their Virulence to Promote Adaptation to the Host

**DOI:** 10.3390/toxins11030135

**Published:** 2019-03-01

**Authors:** Lorena Tuchscherr, Christine Pöllath, Anke Siegmund, Stefanie Deinhardt-Emmer, Verena Hoerr, Carl-Magnus Svensson, Marc Thilo Figge, Stefan Monecke, Bettina Löffler

**Affiliations:** 1 Institute of Medical Microbiology, Jena University Hospital, 07747 Jena, Germany; Christine.Poellath@med.uni-jena.de (C.P.); Anke.Siegmund@med.uni-jena.de (A.S.); Stefanie.Deinhardt-Emmer@med.uni-jena.de (S.D.-E.); Verena.Hoerr@med.uni-jena.de (V.H.); Bettina.Loeffler@med.uni-jena.de (B.L.); 2 Center for Sepsis Control and Care (CSCC), Jena University Hospital, 07747 Jena, Germany; 3 Applied Systems Biology, Leibniz-Institute for Natural Product Research and Infection Biology, 07745 Jena, Germany; Carl-Magnus.Svensson@hki-jena.de (C.-M.S.); Thilo.Figge@hki-jena.de (M.T.F.); 4 Faculty of Biological Sciences, Friedrich Schiller University Jena, 07743 Jena, Germany; 5 Leibniz Institute of Photonic Technology (IPHT), 07745 Jena, Germany; stefan.monecke@leibniz-ipht.de; 6 Institute of Medical Microbiology and Hygiene, Medical Faculty Carl Gustav Carus, 01307 Dresden, Germany

**Keywords:** *S. aureus*, low cytotoxic strains, chronic infection

## Abstract

*Staphylococcus aureus* colonizes epithelial surfaces, but it can also cause severe infections. The aim of this work was to investigate whether bacterial virulence correlates with defined types of tissue infections. For this, we collected 10–12 clinical *S. aureus* strains each from nasal colonization, and from patients with endoprosthesis infection, hematogenous osteomyelitis, and sepsis. All strains were characterized by genotypic analysis, and by the expression of virulence factors. The host–pathogen interaction was studied through several functional assays in osteoblast cultures. Additionally, selected strains were tested in a murine sepsis/osteomyelitis model. We did not find characteristic bacterial features for the defined infection types; rather, a wide range in all strain collections regarding cytotoxicity and invasiveness was observed. Interestingly, all strains were able to persist and to form small colony variants (SCVs). However, the low-cytotoxicity strains survived in higher numbers, and were less efficiently cleared by the host than the highly cytotoxic strains. In summary, our results indicate that not only destructive, but also low-cytotoxicity strains are able to induce infections. The low-cytotoxicity strains can successfully survive, and are less efficiently cleared from the host than the highly cytotoxic strains, which represent a source for chronic infections. The understanding of this interplay/evolution between the host and the pathogen during infection, with specific attention towards low-cytotoxicity isolates, will help to optimize treatment strategies for invasive and therapy-refractory infection courses.

## 1. Introduction 

*Staphylococcus aureus* is a very versatile pathogen that often colonizes the epithelial surfaces of healthy individuals [1,2]; but it is also the most common pathogen of the bloodstream, surgical side, and bone infections, which are often complicated, with several sites of metastatic foci, and the development of chronic infections [3]. In addition, many cases of staphylococcal bacteremia appear to have an endogenous source, since they originate from strains in the nasal mucosa [4]. The diversity of infections that are associated with *S. aureus* is due to the multiple virulence factors and its adaptation to different environments in the human host. This particular adaptation facilitates the bacteria’s survival in the host, and their evasion of the host immune system [5]. 

Recently, we described that *S. aureus* passes different stages of infection through the use of a complicated gene regulatory network [6]. To establish an infection, *S. aureus* displays defined virulence factors, including adhesive surface proteins (adhesins) and toxic compounds that act in concert to destroy the host tissue and to resist the host defense system^2^. In particular, the quorum-sensing system accessory gene regulator (Agr) enhances the expression of toxins, e.g., α-hemolysin/α-toxin (hla), and other secreted cytotoxic factors, e.g., phenol-soluble modulins (PSMs), whereas the alternative sigma factor B SigB (σ^B^) modulates stress responses and promotes *S. aureus* persistence [7,8]. During bacteremia or sepsis, the bacteria need to survive within the bloodstream, to defend against immune cells. Notably, secreted pore-forming toxins, such as *hla*, cause inflammation and contribute to sepsis development [9]. To commence an infection in the host tissue, e.g., bone tissue, bacteria need to adhere to the host structures, such as the extracellular matrix or the host cells. For this, *S. aureus* expresses various surface proteins with adhesive functions, such as fibronectin-binding proteins (FnBPs) [10]. After the infection is settled, the bacteria invade and adapt to the host tissue for persistence and escape from the host immune system, which is mainly mediated by the upregulation of SigB [6]. The intracellular location most likely represents a shelter against many antibiotics, and against the host immune defense system; this causes severe clinical problems in diagnosis and treatment. Chronic infections have been associated with an altered bacterial phenotype, the so-called “small colony variants” (SCVs). SCVs are adapted phenotypes with a reduced metabolism, which enables the bacteria to persist for long-lasting periods [11,12]. However, bacterial adaptation requires the fine-tuning of virulence factors [13]. Yet, the bacterial virulence and its relation to the type of infection are still under discussion. On the one hand, several studies have affirmed that highly virulent and toxin-producing strains cause severe infections [14,15]. On the other hand, a recent study did not find an association between elevated toxicity and the severity of infections [16]. Additionally, some authors have demonstrated that many staphylococcal isolates from invasive diseases have Agr dysfunctions that impair the production of toxins [17,18]. 

In this study, we investigated the role of staphylococcal strains with high and low capacities for inducing host cell death (cytotoxicity). *S. aureus* strains from nasal colonization, endoprosthesis infection, hematogenous osteomyelitis, and sepsis were collected and characterized by genotypic analysis, functional assays, and infection models. Knowing the functions and the interplay between high and low cytotoxic strains will help us to improve the therapeutic treatment and clearance of persistent staphylococcal infections. 

## 2. Results

### 2.1. The Genetic Analysis of Staphylococcal Isolates Reveals Only Minor Differences in Bacterial Origin 

To investigate the virulence of staphylococcal strains from different pathologies, 47 isolates were collected: 12 from nose swabs from healthy individuals (nasal colonization), 12 from orthopedic endoprosthesis infections, 13 from patients with hematogenous osteomyelitis, and 10 from sepsis patients. The main clinical characteristics of all of the patients are summarized in Table S1. The clonal complex affiliations are summarized in Table S2. All of the isolates (four MRSA and 43 MSSA) were analyzed with StaphyType DNA microarrays (Alere Technologies GmbH, Jena, Germany), facilitating the detection of specific genes, as well as their assignment to clonal complexes (CCs). Isolates belonged to a total of 13 different CCs. Although the small sample size excludes a statistically robust evaluation of the relationship between the virulence gene carriage and the clinical outcome, the carriage of some genes was higher, or not present, in some groups (Tables S2 and S3). 

The rate of MRSA was not significantly elevated in one of the groups. The distribution of CCs in the defined groups was very diverse, as every group contained at least five different CCs. The most common CC for isolates with prosthetic origin was CC7 (33.3%) and CC5 (25%). Interestingly, CC7 was highly present in the prosthetic group, but not in other groups (*p* = 0.007, Table S2). Isolates from blood infections, such as hematogenous osteomyelitis and sepsis, showed similar distributions of CCs, where the CC22 was the most abundant (*p* = 0.019, Table S2). Moreover, CC45 was highly prevalent among sepsis isolates. In general, the clonal population structure of study isolates was similar to the one observed in another larger study in Germany [19] (Table S2). 

The complete microarray hybridization data are provided as a Supplementary File (Table S4). The main characteristics of all isolates are summarized in Table S3. The presence of certain genes was significant in some groups, as outlined below. The enterotoxin gene cluster *egc* was present in more than 50% of the isolates of all groups other than prostheses infections (33.3%). Interestingly, the enterotoxin P gene (*entP/seP*) was significantly more common in the prosthetic group than in other groups (Table S3). The hemolysin genes *hla*, *hlb*, and *hld* were present in all isolates. The leukocidin genes *lukF/S-hlgA* were present in all strains, and only one strain from nasal colonization was positive for *lukF/S-PVL*. Yet, genes for *lukD/lukE* were significantly more common in isolates from prosthesis infections than in isolates from nasal colonization and sepsis (Table S3). Genes related to various enzymes and other virulence factors showed a heterogeneous distribution (Table S3). The aureolysin (*aur*), glutamyl endopeptidase (*sspA/B*), the staphylococcal complement inhibitor (*scn*) and staphylokinase (*sak*) genes were found in all isolates. The gene for chemotaxis inhibitory protein (*chip*) was less frequent among prosthesis isolates (25%) in comparison with the other groups (60–80%). Genes encoding serine proteases (*splA/B*) were more prevalent in the bone infection groups than in isolates from sepsis and nasal colonization. Additionally, serin protease E (*splE*) was rarely present in the sepsis group (Table S3). The gene encoding *ssl8* was more prevalent in the prosthetic infection group than in nasal colonization, hematogenous osteomyelitis, and sepsis (Table S3). 

Genes encoding the capsule (*cap5/8*) and biofilm (*icaA-C*) were present in all isolates (Table S3). *cna* was less commonly present in the prosthetic infection group (Table S3). Interestingly, the presence of *ebh* was significantly reduced in isolates from hematogenous osteomyelitis (Table S3). All isolates harbored the genes encoding *ebh, ebps, eno, fib, fnbA, map, sdrC-, sdrD*, and *vwb*. Conversely, the presence of *fnbB* was reduced in the group of hematogenous osteomyelitis (Table S3).

Taken together, our results indicate that similar patterns can be found in hematogenous osteomyelitis and sepsis isolates. Despite some differences in the presence of certain genes between the analyzed groups of staphylococcal isolates, no clear genetic profile was associated with the focus of infection, and focus could be determined (Table S3). 

### 2.2. High- as Well as Low-Cytotoxicity Strains are Equally Distributed among Isolates from Different Bacterial Origins

**Figure 1:**
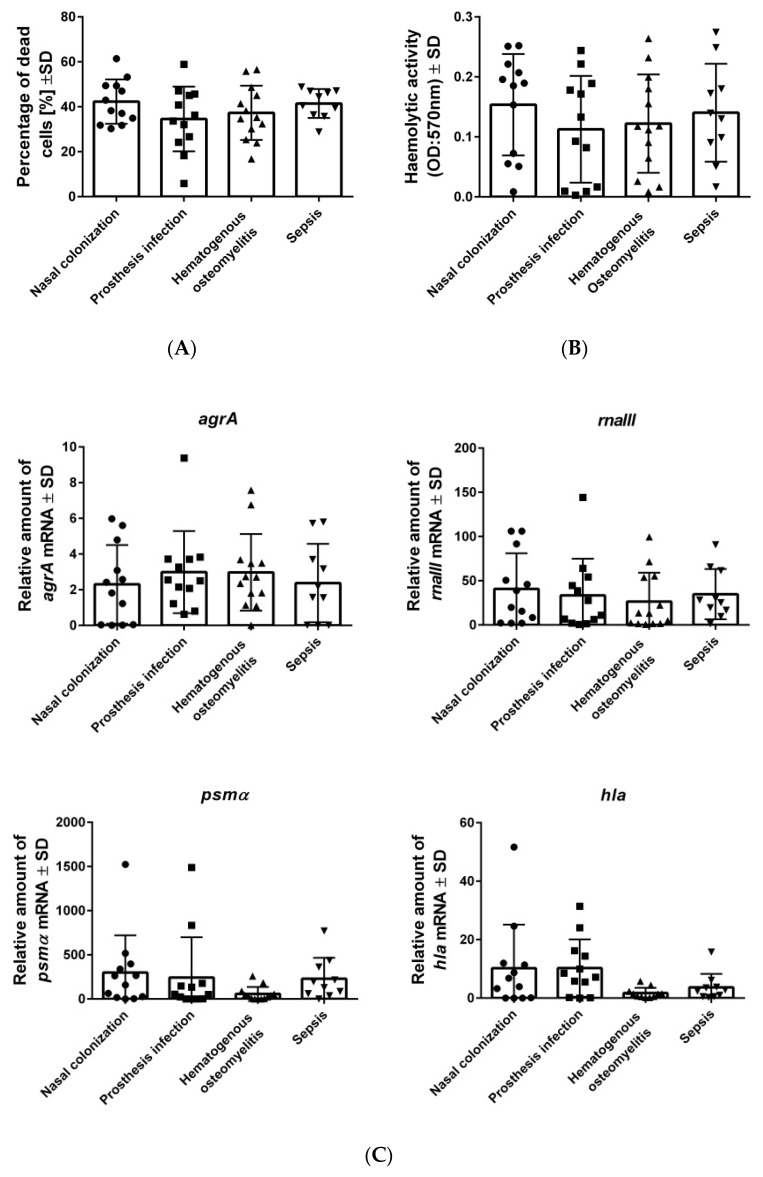
High- and low-cytotoxic strains are similarly distributed among *S. aureus* isolates from different origins. (**A**) Osteoblasts were infected with clinical strains of *S. aureus*, and the percentage of dead cells was measured after 24 h post-infection by staining with propidium iodide and quantification by flow cytometry. (**B**) Hemolysis was measured by detecting the release of hemoglobin from sheep blood erythrocytes (OD: 570 nm) post-infection with *S. aureus* strains. (**C**) Gene expression of *S. aureus* strains. All strains were cultivated in BHI for 4 h (*agrA, rnaIII*) and 6 h (*psmα, hla*) respectively. The RNA was isolated and transformed to complementary DNA (cDNA), in order to perform qPCR. The expression of the gene *gyrB* was used as a reference. The bars and whiskers represent the means ±SD of three independent experiments in duplicate. The differences between all of the groups of isolates were analyzed by a one-way ANOVA test, with Tukey’s multiple comparisons test.

To determine whether the described genetic differences (Table S3) resulted in specific virulence behaviors, further functional assays were performed (Figure 1). The growth curves did not reveal any substantial differences between the isolated strains (Figure S1).

Initially we focused on the functional characteristics that are important for the spreading and settling of infections, such as cytotoxicity, hemolysis, and the expression of related virulence factors (Figure 1). During the first steps of infection, *S. aureus* induces host cell destruction through the expression of membrane disruption toxins, such as α-toxin (*hla*) and phenol-soluble modulins (PSMs) (*psmαβ*) to destroy host tissues, and to favor bacterial spreading [20]. To investigate the cytotoxic capacities of all staphylococcal strains, we analyzed cell death induction in osteoblasts (cytotoxicity), as well as the lysis of erythrocytes (hemolysis) (Figure 1A,B). A broad range of values was observed in each analyzed group in all our assays, but no significance differences based on isolate origins were found. 

To analyze the toxins related to cell death, the expression of *agrA* (accessory gene regulator from the Agr quorum sensing system) and *rnaIII* (RNA effector molecule of this quorum system system), *psmα*, and *hla* were analyzed (see Mat. and Methods, Figure 1C). No differences were observed for *agr, rnaIII*, *and psmα*, regarding the bacterial origin. Only the *hla* expression was up-regulated in the prosthesis infection group, in comparison to strains that were isolated from hematogenous osteomyelitis. In general, a broad distribution of the high and low values was detected in all groups, independent of their bacterial origin.

**Figure 2:**
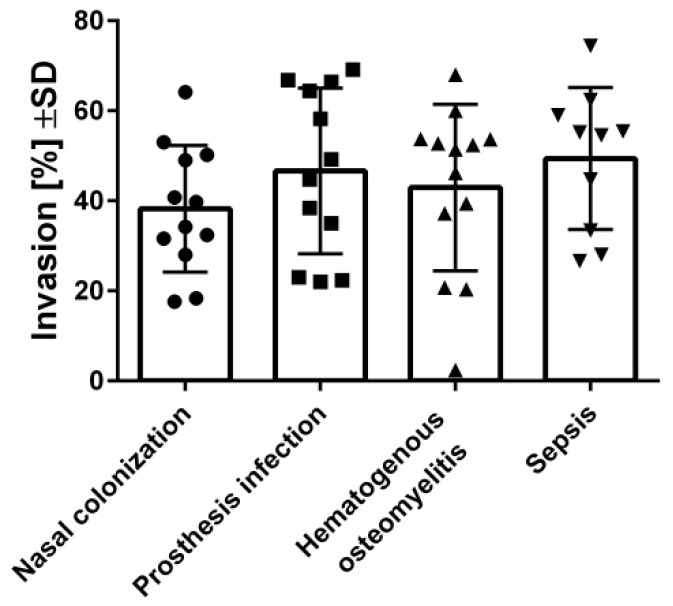
The internalization of *S. aureus* into osteoblasts was non-related to the origin of each isolate. Osteoblasts (hFOB 1.19) were infected with FITC-stained *S. aureus*, and the intracellular bacteria were quantified by flow cytometry. Bars and whiskers represent the means ±SD of at least three independent experiments in duplicate. The differences between all of the groups of isolates were analyzed by a one-way ANOVA test with Tukey’s multiple comparisons test.

After the acute phase when the bacteria induce cell destruction, *S. aureus* can invade host cells to escape from the host immune response. Thus, the invasion of *S. aureus* by osteoblasts was investigated (Figure 2). Similarly to the cytotoxic assays, high and low invasive isolates were found in all analyzed groups of isolates, which was unrelated to their bacterial origin. 

**Figure 3:**
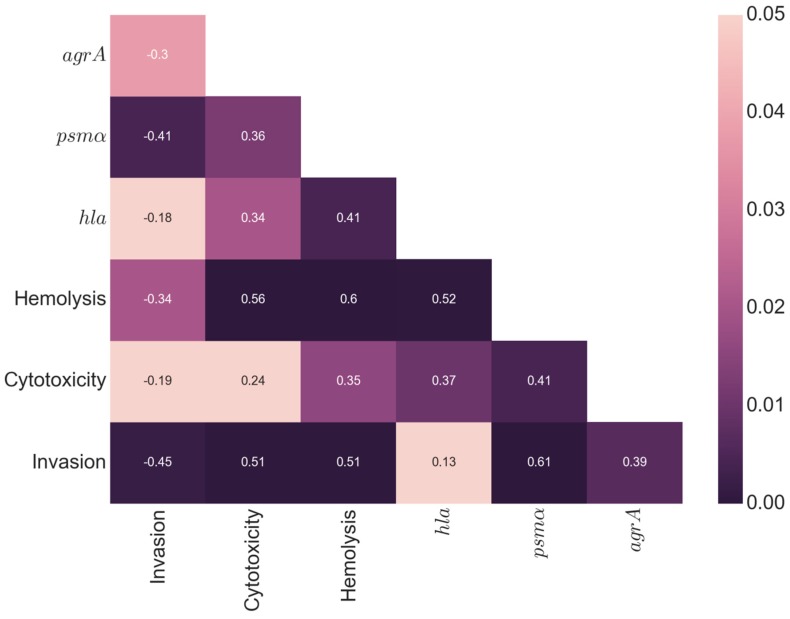
Correlation analysis. Spearman’s correlation coefficients for all of the measured parameters. Significance testing of correlations was done by computing a two-sided *p*-value, assuming a t-distribution with two degrees of freedom. The values in the heat map are the pairwise correlation coefficients (*r*-values) and the colors indicate the significance level of the correlation coefficient (*p* values). Highly significant *p*-values are dark purple, and lighter colors indicate decreasing significance.

Next, we analyzed the pairwise correlation between all measurements, by calculating Spearman’s correlation coefficient (Figure 3). A positive correlation was found between cytotoxicity and hemolysis (*p* = 0.01; *r* = 0.36) indicating an overlap of factors for both processes. Interestingly, a negative correlation was found between cytotoxicity/hemolysis and invasion, suggesting that both processes are inversely correlated (*p* = 0.05; *r* = −0.3) (*p* = 0.004; *r* = −0.41). As expected, a positive correlation was found between cytotoxicity and the expression of *hla* (*p* = 0.02; *r* = 0.34); *psmα* (*p* < 0.001; *r* = 0.56) and *rnaIII* (*p* < 0.001; *r* = 0.51). The induction of hemolysis is correlated with the expression of *hla* (*p* = 0.004; *r* = 0.41); *psmα* (*p* < 0.001; *r* = 0.6), *agrA* (*p* = 0.02; *r* = 0.35) and *rnaIII* (*p* < 0.001; *r* = 0.51). A positive correlation was found between the expression of *rnaIII* and *psmα* (*p* < 0.001; *r* = .61) and *agrA* (*p* < 0.01; *r* = 0.39) as previously described [21]. Even though the correlation *p*-values were significant, the moderate magnitude of the correlation coefficients, in a range between 0.1 and 0.6, reflected the large variation in the measurements between the strains.

Cytotoxicity displayed a wide range of values, but it was not significantly different between the types of infection, and in each infection type, isolates from the entire spectrum of cytotoxicity were present (Figure S2). The correlation analysis suggests that highly cytotoxic strains display a strong induction of cell death in general (cytotoxic and hemolysis), a low invasion capacity, and a high expression of *hla*, *psmα*, *rnaIII*, and *agrA*, which enabled the bacteria to spread to different tissues by killing host cells during the acute phase. In contrast, strains with low cytotoxic levels showed reduced rates of cell death, but they achieved a higher degree of host cell internalization compared to highly cytotoxic strains. This internalization is necessary for their escape from the host immune response, and antimicrobial treatment. 

### 2.3. Highly Invasive but Low-Cytotoxicity Strains Persist at High Numbers within Host Cells 

**Figure 4:**
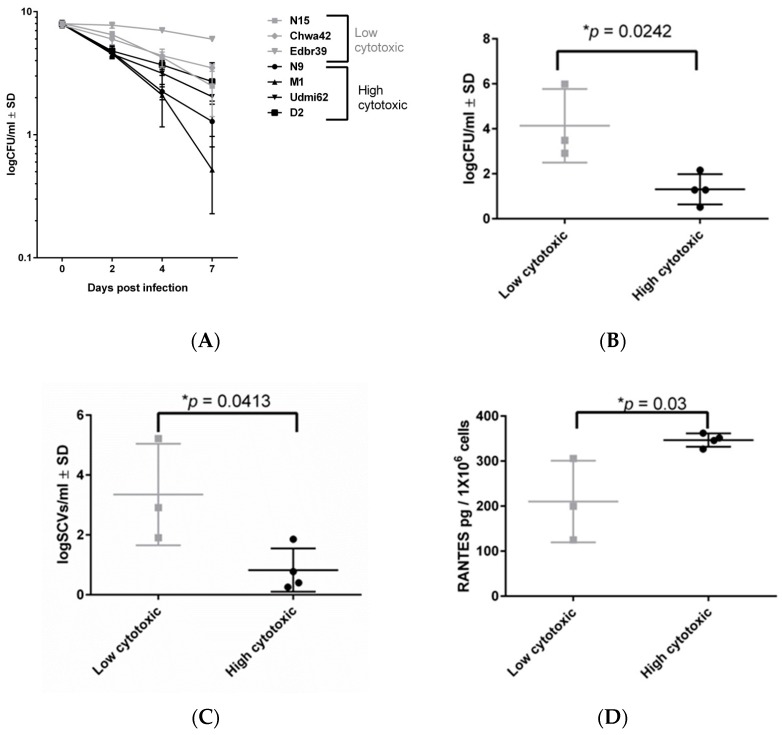
Long-term persistence of low- and high-cytotoxicity strains in osteoblasts. (**A**) Intracellular bacteria recovered from infected osteoblasts for four high cytotoxic strains (black lines) and three low cytotoxic strains (gray lines) as a function of time post-infection. (**B**) Intracellular bacteria recovered from infected osteoblasts with high- and low-cytotoxicity strains at day 7 post-infection. (**C**) SCVs recovered at day 7 post-infection. (**D**) RANTES levels were measured in the cell culture supernatant of infected osteoblasts after 24 h post-infection, using the ELISA test. All results represent the mean ±SD of at least three independent experiments. The difference between the low- and high-cytotoxicity strains was analyzed by the Unpaired *t*-test. (* *p* < 0.05; ** *p* < 0.01; *** *p* < 0.001 and **** *p* < 0.0001)

*S. aureus* can invade its host cells, and it is able to persist intracellularly for long time periods, while down-regulating its metabolism and toxin expression [22]. To investigate the association between virulence and persistence, four highly cytotoxic and three low-cytotoxicity strains were selected according the cytotoxic and hemolysis values (Table S5). The persistence was analyzed by infecting osteoblasts for up to seven days (Figure 4A). We found that both high- and low-cytotoxicity strains were able to survive within the host cells (Figure 4A). Strikingly, the highly cytotoxic strains had reduced intracellular survival, compared to the low-cytotoxicity strains after seven days (Figure 4B). Moreover, the low-cytotoxicity strains survived in higher numbers, and induced a higher rate of SCV formation (Figure 4C). Next, the inflammatory response in osteoblasts was investigated by the determination of CCL5 (RANTES) by ELISA after 24 h of infection (Figure 4D). All highly cytotoxic strains induced more inflammation than the low-cytotoxicity strains. Taken together, our results show that staphylococcal low-cytotoxicity strains promote long-term intracellular persistence by inducing a reduced inflammatory response and cell death, which contributes to their survival within host cells in higher numbers.

### 2.4. Low-Cytotoxicity Strains can Persist in High Numbers in a Murine Sepsis Model

**Figure 5:**
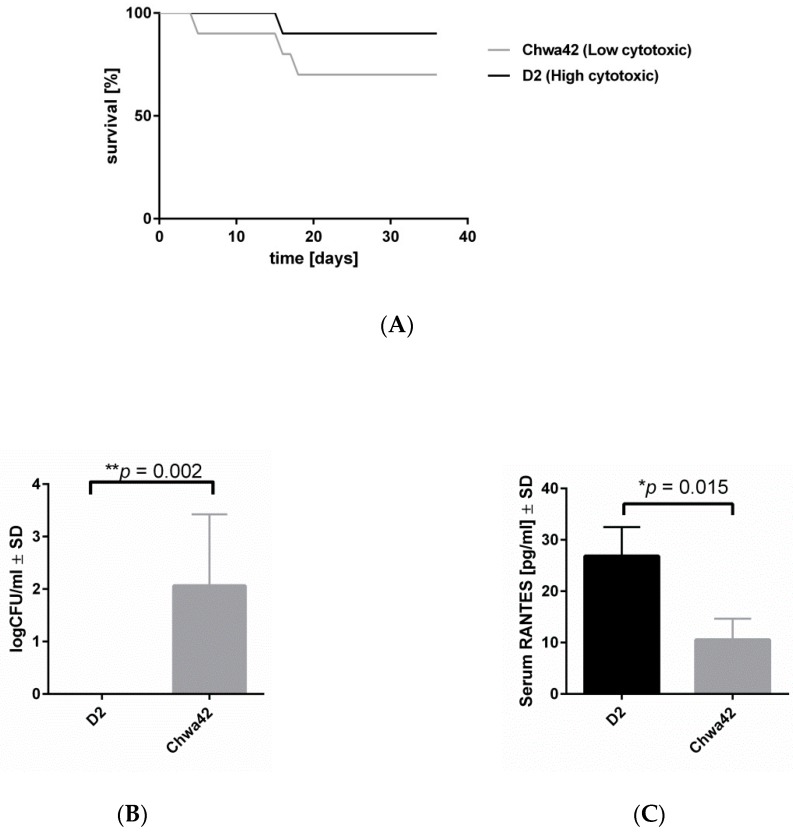
A highly cytotoxic strain induced high inflammation and fast clearance by the host in the mouse sepsis model. C57/BL6 mice were infected with Chwa42 (low cytotoxic) or D2 (high cytotoxic) strains for six weeks. (**A**) Survival curves of the infected mice with Chwa42 (n = 8) and D2 (n = 10). No differences were observed according to the long-rank (Mantel–Cox) test. (**B**) The bacterial loads within the tibiae were analyzed after six weeks post-infection. The bar and whiskers represent the mean ± SD. Statistical analysis was performed with an unpaired *t*-test comparing the bacterial load in the tibiae (** *p* = 0.0021). (**C**) RANTES levels in the serum were measured after three days post-infection. A Statistical analysis was performed, using the unpaired *t*-test, comparing the abundance of RANTES in the serum (* *p* = 0.0015).

To test whether bacterial virulence patterns affect the development of infection, we performed our hematogenous osteomyelitis model [23]. Mice were infected with a low-cytotoxicity (Chwa42; Table S5) or a high-cytotoxicity *S. aureus* strain (D2; Table S5) and we analyzed the bacterial loads in the bones after six weeks post-infection. No difference in murine survival was observed after infection between both strains (Figure 5A). The bacterial loads in bones after six weeks post-infection revealed a higher persistence and less efficient clearance of the low-cytotoxicity strain Chwa42, in comparison to the highly cytotoxic strain D2 (Figure 5B). Furthermore, low inflammatory levels of CCL5 (RANTES) was measured in the serum, from mice infected with the low-cytotoxicity strain Chwa42, whereas the highly cytotoxic strain D2 enhanced the release of the indicated chemokine (Figure 5C). Taken together, our results suggest that low-cytotoxicity strains are able to maintain infection by evading host clearance.

## 3. Discussion 

*S. aureus* is a versatile microorganism that causes a diverse array of infections. For different pathologies connected to *S. aureus* a controversial link between virulence and clinical outcome has been discussed [16,24,25]. Bacterial virulence is defined as the capacity for a pathogen to cause disease in the host [26] The virulence is influenced by a diversity of ecological factors, such as the surrounding environment during the course of infection, which either enhances or reduces the expression of specific virulence factors [5]. Thus, we assumed as a first hypothesis of our work, that different environments/origins of infection may determine the virulences of the staphylococcal isolates. 

To investigate the associations between genes for virulence and infection types, we collected staphylococcal strains from nasal colonization, and from patients suffering from prosthesis infection, hematogenous osteomyelitis, and sepsis. By genotypic analysis, we found only a few genes with different prevalence in the respective groups. Moreover, some differences were related to clonal complex affiliations, and not to the type of infection. 

To analyze whether the genetic characteristics affected the interactions between *S. aureus* and the host, we performed several functional assays related to the course of infection. During the first few steps of infection, *S. aureus* expressed several toxins and exoenzymes to induce tissue destruction, and to facilitate bacterial dissemination [27]. Thus, we investigated cell death induced by staphylococcal isolates from all groups on osteoblasts (cytotoxicity), and their capacity to lyse erythrocytes (hemolysis). Furthermore, the expression of main toxins, such as *hla* and *psmα*, as well as the expression of virulence global regulators such as *agr* and *rnaIII* were measured. We found a wide range in the cytotoxic capacity between the different strains, but we could not correlate cytotoxicity or hemolysis with the different infection types. By studying our bacterial collections from different origins, we demonstrated that low-cytotoxicity strains were present in all of the analyzed groups, including the healthy nasal carriers, indicating that low- and not only highly destructive strains are part of the pathogenic process of *S. aureus*. In line with these results, recent studies have demonstrated a higher propensity for low cytotoxic isolates to cause bacteremia, which can be explained by evolutionary trade-offs and bacterial fitness [16]. Toxins must not only be considered as tissue-destructive agents, but they are also involved in many functions, such as activating or manipulating the host immune system, and biofilm and colonization processes. Supporting the multiple functions of toxins besides tissue destruction, we also found a positive correlation between cytotoxicity and toxin expression [28]. Consequently, the role of cytotoxicity for the long-term survival of bacteria within the host is highly complex, and it needs to be tightly regulated to favor bacterial persistence. 

Following tissue destruction and bacterial spreading, *S. aureus* invades the host cell to settle an infection. Thus, the invasiveness of staphylococcal isolates was analyzed by infecting osteoblasts and measuring the number of intracellular bacteria. Again, no differences were found to be related with the bacterial origins, but here also, a wide distribution between the highly and low-invasive strains was found in all of the analyzed groups. 

Furthermore, positive and significant correlations were found between cytotoxicity as well as hemolysis, and the expression of *hla, psmα, agr*, and *rnaIII*, as these genes are largely responsible for the cytotoxic capacity of *S. aureus* [27]. By contrast, most of these parameters showed a significant but negative correlation with invasiveness. According to our functional assays, we defined highly cytotoxic strains that trigger high cell death, as having a high expression of *hla, psmα, agr*, and *rnaIII*, but a low degree of invasiveness. In contrast, the low-cytotoxicity strains were characterized by high invasiveness and a low expression of toxins. 

To analyze the roles of high- and low-cytotoxicity strains during the course of infection, we investigated the ability for the long-term persistence of selected strains from each group. Furthermore, the appearance of SCVs was analyzed as a sign of adaptation and persistence. It is well-known that the SCVs can be induced/formed, or selected under stress conditions, such as intracellular locations [11,22]. Interestingly, high- and low-virulence strains were able to survive within host cells for up to seven days and form SCVs, indicating that the persistence mechanisms and SCV formation are general features of all *S. aureus* strains. Nevertheless, the low-cytotoxicity strains persisted in higher numbers, and presented more SCVs than the highly cytotoxic strains. These results indicate that low-cytotoxicity strains are better adapted for long-term persistence and survival within the host. In particular, in our murine model, we found that highly cytotoxic strains induced greater immune responses than low-cytotoxicity strains, which contributed to clearing the infecting bacteria. Consequently, the host is often not able to fully clear infections by low-cytotoxicity strains, which can turn into chronic and therapy-refractory disease courses.

**Figure 6:**
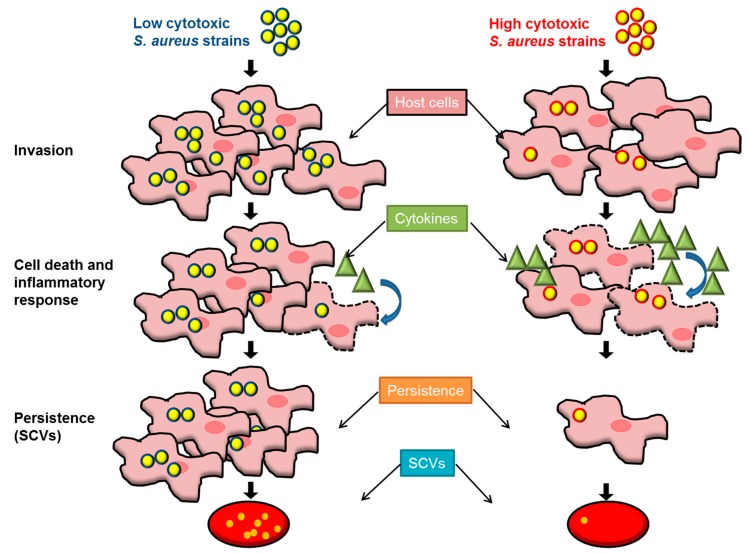
Low-cytotoxicity strains are able to persist in higher numbers, and they represent a bacterial reservoir for further infections. Low-cytotoxicity and highly cytotoxic staphylococcal strains are present in every staphylococcal infection, but they promote different mechanisms of infection. Highly cytotoxic strains display low invasion capacities and a high expression of toxins such as *hla, psmα* and others regulated by *rnaIII* and *agrA*, which promote the release of high amounts of cytokines. In this scenario, the bacteria are able to kill the host cells. Thus, only a few cells contain bacteria which can switch to SCVs. This pathway allows the bacteria to disseminate into other tissues, and this takes place during the acute phase. On the contrary, low-cytotoxicity strains tend to cause lower rates of cell death, but they achieve a higher level of host cell internalization compared to highly cytotoxic strains. Due to their low expression of virulence factors, the low–cytotoxicity strains are able to maintain the integrity of the host cells, and higher numbers of bacteria can switch to SCVs, favoring the silent persistence of *S. aureus*.

These results are in line with recently published work demonstrating that genetic and phenotypic diversity favor infection development and the survival of the bacteria within the host [16,24,29,30]. Bacterial diversity must not be neglected as a virulence strategy. On one hand, highly virulent bacteria are able to attack and spread within the host, and in a population of hosts. On the other hand, less virulent bacteria can easier escape from the host immune system and survive within the host organism, and/or spread to other hosts (Figure 6). Apparently, different invasive infections (such as bone, foreign material infections, and sepsis) follow similar pathogenic courses, as we could not detect significant differences between strains isolated from different foci, nor in their degree of virulence. Moreover, the evolutionary success of *S. aureus* may be caused by heterogeneity within bacterial populations, and thus by its ability to vary, to evolve and to adapt, allowing it to induce a wide range of different types of infection in a range of different host species. It is already known that diversity within a bacterial population is an advantage for survival under environmental changes, and this has already been demonstrated for other bacterial species [29,31,32]. Furthermore, the low-cytotoxicity strains were present, not only in groups of different infections, but also in healthy nasal colonization. Thus, low-cytotoxicity strains are already positively selected during colonization, because they enhance bacterial fitness, resulting in a large remainder population that can turn into SCVs. The SCVs, the dormant phenotype, are able to persist under non-favorable conditions, such as low nutrition, high oxidative stress, and antibiotic treatment. 

In summary, our results emphasize the importance of low-cytotoxicity strains in different pathologies induced by *S. aureus*. The low-cytotoxicity strains can adapt to the host, are poorly eliminated, and remain in higher numbers to select/form SCVs as a reservoir for chronic infection courses. These characteristics enhance the bacterial transmissibility, fitness, and antimicrobial tolerance. The understanding of this interplay/evolution between the host pathogen during infection, with specific attention on low cytotoxic isolates, will help us to optimize the treatment strategies for invasive and therapy-refractory infection courses. 

## 4. Material and Methods

### 4.1. Collection of S. aureus Isolates and Definitions

The bacterial isolates were obtained from the Jena University Hospital. Identification procedures were performed in our lab by Vitek (Biomerieux, Germany). The corresponding patients resided in Thuringia and the adjacent federal states (Saxony-Anhalt, Bavaria and Hessen). The isolates were grouped into different categories: sepsis, hematogenous osteomyelitis, prosthesis (orthopedic patients with endoprosthesis), and nasal isolates from healthy persons. The main clinical characteristics are summarized in Table S1. 

Sepsis was defined according to a definition on the basis of the quick Sepsis-related Organ Failure Assessment score (qSOFA) [33]. Patients belonging to the group of hematogenous osteomyelitis were characterized by a bone infection and an *S. aureus*-positive blood culture. The prosthesis groups were isolates from patients with prosthesis infections and a negative score for the blood culture. The nasal isolates were taken from a larger collection from our institute, and have previously been investigated [34].

### 4.2. Genotypic Characterization 

The *S. aureus* isolates were genotyped with the Alere StaphyType DNA microarray (Alere Technologies GmbH, Jena, Germany). This array allowed for their assignment to clonal complexes, and the detection of 170 genes with their allelic variants.

### 4.3. Growth Curve and Generation Time 

For growth curves, overnight cultures from *S. aureus* strains in BHI were diluted to OD_578nm_ = 0.05, and incubated at 37 °C with shacking (160 rpm). Turbidity (OD_578nm_) was measured every 15 min for 17 hours (hr). The growth rate (μ, growth speed) and the generation time (g) for each strain used in this study (Figure S1) were calculated according the standard formula by Madigan et al. [35].

### 4.4. Measurement of Haemolysis

The hemolysis assay was performed by using the protocol described previously [6]. 

### 4.5. Cell Death and Invasion Assay in Osteoblasts

The human osteoblast cell line hFOB 1.19 (ATCC CRL-11372) was cultured according to the manufacturer’s protocol. The invasion assay was performed by flow cytometry (BD Accuri™ C6), as described previously [36], but only raw values were used. The rate of cell death in the hFOB 1.19 cells was determined by measuring the uptake of propidium iodide (PI), as described previously [37].

### 4.6. Long-Term Persistence

The human osteoblasts cell line hFOB 1.19 (ATCC CRL-11372) was cultivated, following the manufacturer’s indications, and it was infected with different *S. aureus* strains (Table S5). Briefly, osteoblasts cells were infected with a MOI (multiplicity of infection) of 50. After 1.5 hr, cells were washed with PBS, and lysostaphin (20 μg/mL) was added for 30 min, to lyse all extracellular or adherent staphylococci, and then fresh culture medium with Penicillin and streptomycin were added to the cells. The washing, the lysostaphin, and medium-exchange steps were repeated every two days, to remove all of the extracellular staphylococci. To detect live intracellular bacteria at different time points post-infection (p.i.), host cells were lysed in H_2_O, and the number of colony-forming units (CFU) was determined by serial dilutions on blood agar. The colony phenotypes were analyzed by a Colony Counter Shuett (Biosys, Karben, Germany). SCVs were colonies with a diameter < 0.6 mm. 

### 4.7. RNA Isolation and Real-Time PCR

To determine gene expression in the early stationary phase, overnight bacterial cultures were diluted to an OD_578nm_ of 0.05, and incubated at 37 °C with rotation (160 rpm). The time points for the higher expression of each factor were estimated by analyzing the expression of each gene in the different staphylococcal strains used in our lab (SH1000, LS1, 6850, USA300, and selected clinical strains). After 4 h (*agrA*, *rnaIII*) and 6 h (*psmα*, *hla*) of incubation, 1 mL of bacterial suspension was mixed with RNAprotect Bacteria Reagent (Qiagen), centrifuged, and stored at −20 °C. For isolation of RNA, the pellet was mixed with RNApro™ Solution (MP Biomedicals, Eschwege, Hessen, Germany). The mixture was transferred to a Lysing Matrix B tube (MP Biomedical) and homogenized with a FastPrep^®^ (MP Biomedicals) homogenizer. After subsequent centrifugation, the supernatant was used for RNA isolation with the peqGOLD Total RNA Kit (VWR) following the manufacturer’s instructions. DNA was digested with TURBO™ DNase (ThermoFisher Scientific, Darmstadt, Germany). The concentration of the RNA was determined by spectrophotometric analysis with a NanoDrop (ThermoFisher Scientific, Darmstadt, Germany) before reverse transcription to complementary DNA (cDNA) (qScript cDNA SuperMix, Quantabio, Beverly, MA, USA). The cDNA was analyzed with QuantiNova SYBR Green PCR Kit (QIAGEN, Hilden, Germany) in a Rotor-Gene Q (QIAGEN) thermocycler. The reaction mixtures were incubated for 15 min at 95 °C, followed by 40 cycles of 15 s at 95 °C, 30 s at 55 °C, and 30 s at 72 °C. The primers used are described in Table S6. Fold-changes in expression were calculated by the Pfaffl equation [38]. The *gyrB* gene was used as a reference. 

### 4.8. Murine Sepsis Model

Our murine sepsis model was performed as described before [23]. C57BL/6 10-week-old female mice were obtained from the central laboratory animal facility of the Jena University hospital. The animals were maintained according to institutional guidelines in individually ventilated cages, and were given food and water ad libitum. Mice were inoculated with 1 × 10^6^ CFU of *S. aureus* in 200 µL of PBS, via a lateral tail vein, and sacrificed by CO_2_ asphyxiation after three days (acute) and six weeks (chronic) post-infection. For the enumeration of bacteria in the tibiae of infected mice, homogenates were prepared in PBS and plated in 10-fold serial dilutions on blood agar. 

### 4.9. Release of the Chemokine RANTES

The cell supernatants from the long-term persistence experiment were analyzed with RANTES human Instant ELISA™ (ThermoFisher Scientific). 

Serum samples from infected mice were taken after three days post-infection, and analyzed by RANTES Mouse Instant ELISA™ (ThermoFisher Scientific).

### 4.10. Ethical Permissions

The ethical approval for the bacterial strains and the patients’ data was approved by the local ethical committee of the University of Jena 4874-07/16 and 4449-06/15 (Thüringen, Jena). The murine sepsis model infection model was conducted in accordance with the recommendation and guidelines of the German regulations of the Society for Laboratory Animal Science 22-2684-04-02-006/15 and 22-2684-04-02-046/16 (Thüringen, Jena).

### 4.11. Statistics

The distribution of clonal complexes and the frequency of selected genes among all groups were analyzed by Fisher’s exact test. The functional assays and statistical analyses of gene expression were performed by using GraphPad Prism version 4.00 (Graphpad, La Jolla, CA, USA). The normality of the distribution was analyzed with the D’Agostino & Pearson omnibus, and the Shapiro–Wilk normality test. Additionally, all of the results were tested for outliers with the ROUT test. An unpaired *t*-test was used when two groups were compared. Multiple groups were compared by and ordinary one-way ANOVA test, followed by Tukey’s multiple comparisons test. According to the *p*-values, the differences were: either not significant (ns, *p* > 0.05); or significant (* *p* < 0.05; ** *p* < 0.01; *** *p* < 0.001 and **** *p* < 0.0001). The pairwise correlations between all of the measured parameters were performed by calculating Spearman’s correlation coefficient and a two-sided *p*-value was used. A t-distribution with two degrees of freedom for testing the significance of the coefficients was assumed. Coefficients and *p*-values were both calculated using the Python library SciPy [39]. The difference between both survival mice curves was analyzed by the long-rank (Mantel Cox) test.

## Supplementary Materials

The following are available online at https://www.mdpi.com/2072-6651/11/3/135/s1, Figure S1: Growth dynamics of *S. aureus* isolates, Figure S2: Cytotoxicity of *S. aureus* isolates, Table S1: Clinical characteristics of all patients, Table S2: Distribution of clonal complexes amongst staphylococcal isolates from nasal colonization, prosthesis infection, hematogenous osteomyelitis, and sepsis, Table S3: Frequencies of selected genes in the defined isolate groups, Table S4: Alere complete results, Table S5: Main characteristics of selected strains for long-term cell culture and the mouse sepsis model, Table S6: Primers used in this study.
