# Supplementary material for: Clinical S. aureus Isolates Vary in Their Virulence to Promote Adaptation to the Host
Source: Toxins (Basel). 2019 Mar 1;11(3):135. doi: 10.3390/toxins11030135 (PMC6468552; doi:10.3390/toxins11030135)
Supplement: Supplementary file 1 [file toxins-11-00135-s001.zip › toxins-11-00135-s001/toxins-11-00135-s001.pdf]

Supplementary Materials: Clinical *S. aureus* Isolates Vary in Their Virulence to Promote Adaptation to the Host

Lorena Tuchscherr, Christine Pöllath, Anke Siegmund, Stefanie Deinhardt-Emmer, Verena Hoerr, Carl-Magnus Svensson, Marc Thilo Figge, Stefan Monecke and Bettina Löffler

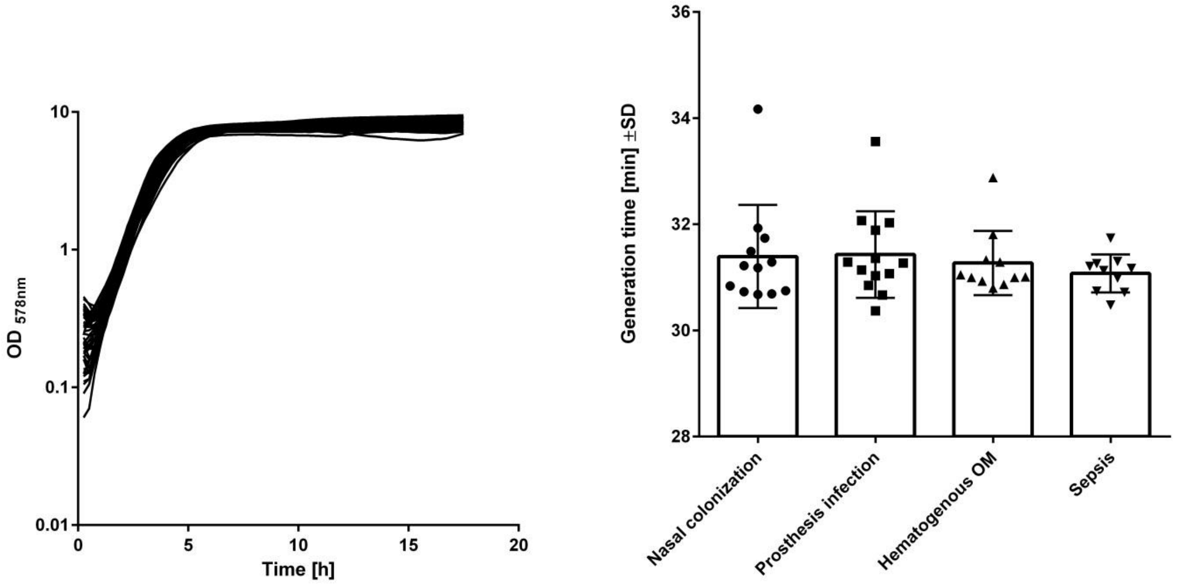

(**A**) (**B**)

Figure S1. Growth dynamics of *S. aureus* isolates.

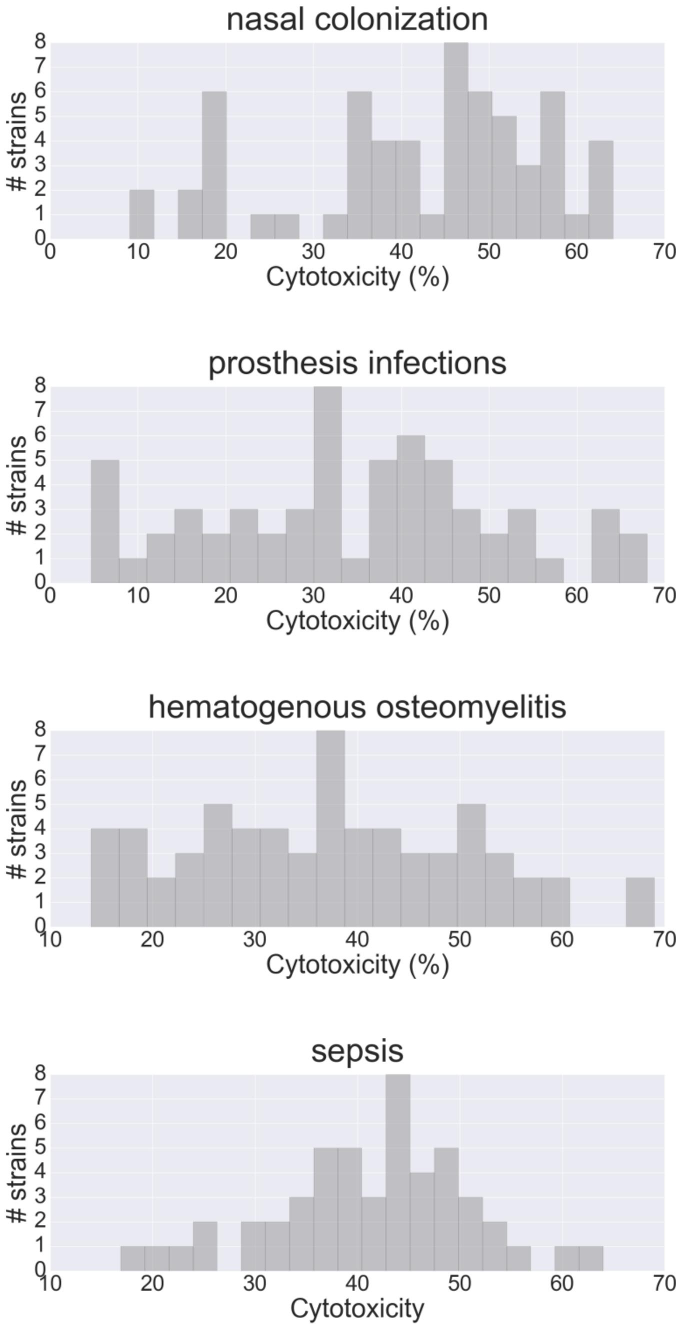

Figure S2. Cytotoxicity of *S. aureus* isolates.

Table S1. Clinical characteristics of all patients.

Characteristics	Nasal Colonization	Prosthesis Infection	Hematogenous Osteomyelitis	Sepsis
**Total Number**	12	12	13	10
**Median Age [years]**	49.08 (range 40–64)	66 (range 33–85)	75.23 (range 47–96)	62.7 (range 53–78)
**Male**	6 (50,0%)[a]	9 (75.0%)	8 (61.5%)	5 (50,0%)
**Case Fatality**	-	0	5 (38.5%)	2 (20.0%)
**Underlying Condition**				
**Immunosupression**	-	2 (16.7%)	3 (23.1%)	3 (30.0%)
**Diabetes Mellitus**	-	2 (16.7%)	5 (38.5%)	7 (70.0%)
**Malignant Disease**	-	0	2 (15.4%)	2 (20.0%)
**Indwelling Devices**	-	7 (58.3%)	2 (15.4%)	1 (10.0%)
**Renal Disease**	-	5 (41.7%)	12 (92.3%)	7 (70.0%)
**Cardiovascular Disease**	-	9 (75.0%)	8 (61.5%)	9 (90.0%)
**Liver Disease**	-	3 (25.0%)	1 (7.7%)	4 (40.0%)
**Origin of Infection/Primary Focus**				
**Unknown**	-	0	3 (23.0%)	0
**Bone**	-	12 (100%)	7 (53.8%)	0
**Wound/Skin**	-	0	1 (7.7%)	2 (20.0%)
**Intravenous Catheter**	-	0	2 (15.4%)	0
**Lung**	-	0	0	6 (60.0%)
**Gastrointestinal Tract**	-	0	0	2 (20.0%)
**Hospital Stays During Last 3 years (in Relation to Infection)**				
**None**	-	3 (25%)	10 (76.9%)	9 (90.0%)
**1**	-	0	2 (15.4%)	0
**≥ 2**	-	9 (75%)	1 (7.7%)	1 (10.0%)
**Median Length of Hospital Stay [days]**	-	60 (range 14–1080)[b]	30 (range 9–180)	17,5 (range 2–360)

a: one sample couldn't be assigned to sex; b: no data available for one isolate from the group of prosthesis infection.

Table S2. Distribution of clonal complexes amongst staphylococcal isolates from nasal colonization, prosthesis infection, hematogenous osteomyelitis and sepsis. The number of isolates are indicated between parentheses.

**Clonal Complex (CC)**	**Nasal Colonization**	**Prosthesis Infection**	**Hematogenous Osteomyelitis**	**Sepsis**	***p* *(Fisher exact test)***
	**(n = 12)**	**(n = 12)**	**(n = 13)**	**(n = 10)**	
**CC1**	0	0	7.7% (1)	10% (1)	0.58
**CC5**	16.6% (2)	25% (3)	0	10% (1)	0.27
**CC6**	8.3% (1)	8.3% (1)	7.7% (1)	0	1
**CC7**	0	33.3% (4)	0	0	0.007
**CC8**	0	16.6% (2)	15.4% (2)	10% (1)	0.62
**CC15**	8.3% (1)	0	15.4% (2)	10% (1)	0.72
**CC22**	0	0	30.8% (4)	30% (3)	0.02
**CC25**	8.3% (1)	0	7.7% (1)	0	1
**CC30**	16.6% (2)	8.3% (1)	0	0	0.38
**CC45**	25% (3)	0	7.7% (1)	30% (3)	0.12
**CC101**	0	0	7.7% (1)	0	1
**CC121**	0	8.3% (1)	0	0	0.72
**CC398**	16.6% (2)	0	0	0	0.16

Table S3. Frequencies of selected genes in the defined isolate groups.

Gene	Product	Nasal colonization	Prosthesis infection	Hematogenous osteomyelitis	Sepsis	*p* (*Fisher test*)
		**Regulation**				
agrI	Accessory gene regulator allele I	58.3% (7)	66.7% (8)	69.2% (9)	70.0% (7)	0.95
agrII	Accessory gene regulator allele II	25.0% (3)	25.0% (3)	15.4% (2)	20.0% (2)	0.93
agrIII	Accessory gene regulator allele III	16.7% (2)	8.3% (1)	7.7% (1)	10.0% (1)	0.93
agrIV	Accessory gene regulator allele IV	0	0	7.7% (1)	0	1
		**Resistance**				
bla	β-lactamase	58.3% (7)	58.3% (7)	46.2% (6)	90.0% (9)	0.18
ermA	Erythromycin / clindamycin resistance gene A	0	16.7% (2)	0	20.0% (2)	0.17
ermB	Erythromycin / clindamycin resistance gene B	0	0	0	0	-
ermc	Erythromycin / clindamycin resistance gene C	0	8.3% (1)	0	0	0.72
fosB	Metallothiol transferase	58.3% (7)	66.7% (8)	53.8% (7)	30.0% (3)	0.42
mecA	Alternate penicillin binding protein 2, defining MRSA	0	16.7% (2)	7.7% (1)	10.0% (1)	0.61
tetEfflux	Transport-/ efflux protein	100% (12)	100% (12)	69.2% (9)	70.0% (7)	**0.03**
tetK	Tetracycline resistance gene K	0	0	7.7% (1)	10.0% (1)	0.58
tetM	Tetracycline resistance gene M	0	8.3% (1)	0	0	0.73
vanA	Vancomycin resistance gene	0	0	0	0	-
		**Enterotoxins**				
sea	Staphylococcal enterotoxin A	16.7% (2)	16.7% (2)	15.4% (2)	0	0.6
seb	Staphylococcal enterotoxin B	8.3% (1)	0	15.4% (2)	0	0.6
sec, sel	Staphylococcal enterotoxin C+L	25.0% (3)	0	15.4% (2)	10.0% (1)	0.33
sed, sej, ser	Staphylococcal enterotoxin D+J+R	0	25.0% (3)	7.7% (1)	20.0% (2)	0.24
see	Staphylococcal enterotoxin E	0	0	0	0	-
egc-cluster	Staphylococcal enterotoxin G+I+M+N+O+U	66.7% (8)	33.3% (4)	53.8% (7)	70.0% (7)	0.29
seh	Staphylococcal enterotoxin H	8.3% (1)	0	7.7% (1)	10.0% (1)	0.89
sek, seq	Staphylococcal enterotoxin K+Q	0	8.3% (1)	7.7% (1)	0	1
tst1	Toxic shock syndrome toxin (TSST)-1	16.7% (2)	0	0	0	0.16
entP	Enterotoxin P	0[b]	41.7% (5) [b]	0	10.0% (1)	**0.003**
		**Hemolysins**				
hla	α-toxin	100% (12)	100% (12)	100% (13)	100% (10)	-
hlb	β-toxin	75.0% (9)	100% (12)	11	80.0% (8)	0.38
hld	δ-toxin	100% (12)	100% (12)	100% (13)	100% (10)	-
		**Leukocidins**				
lukF, hlgA	γ-toxin	100% (12)	100% (12)	100% (13)	100% (10)	-
lukF-PV	Panton-Valentin-leukotoxin	8.3% (1)	0	0	0	0.72
lukD, lukE	Leukocidin D, E component	41.7% (5) [c]	91.7% (11) [c]	61.5% (8)	40.0% (4)	**0.034**
		**Exfollative toxins**				
etA	Exfollative toxin A	8.3% (1)	0	0	0	0.72
etB	Exfollative toxin B	0	0	0	0	-
etD	Exfollative toxin D	8.3% (1)	0	7.7% (1)	0	1
		**Enzymes**				
aur	Aureolysin	100% (12)	100% (12)	100% (13)	100% (10)	-
chp	Chemotaxis inhibitory protein (CHIP)	66.7% (8)	25.0% (3)	61.5% (8)	80.0% (8)	0.06
splA, splB	Serine protease A, B	41.7% (5)[d]	91.7% (11)[d]	61.5% (8)	40.0% (4)[d]	**0.034**
splE	Serine protease E	41.7% (5)	66,7% (8)[e]	46.2% (6)	10% (1)[e]	0.07
sspA, sspB	Glutamyl endopeptidase	100% (12)	100% (12)	100% (13)	100% (10)	-
sak	Staphylokinase	66.7% (8)[f]	100% (12)[f]	84.6% (11)	80.0% (8)	0.17
scn	Staphylococcal complement inhibitor	91.7% (11)	100% (12)	100% (13)	100% (10)	0.72

		**Exopolysaccharides**				
cap5	Capsular polysaccharide 5	41.7% (5)	41.7% (5)	46.2% (6)	50.0% (5)	1
cap8	Capsular polysaccharide 8	58.3% (7)	58.3% (7)	53.8% (7)	50.0% (5)	1
icaA, D, C	Polysaccharide intracellular adhesin	100% (12)	100% (12)	100% (13)	100% (10)	-
		**Adhesins**				
bbp	Bone sialoprotein-binding protein	91.7% (11)	91.7% (11)	100% (13)	100% (10)	0.71
clfA, clfB	Clumping factors A and B	100% (12)	100% (12)	100% (13)	100% (10)	-
cna	Collagen binding adhesin	58.3% (7)	25.0% (3)	61.5% (8)	70.0% (7)	0.15
ebh	Cell wall associated fibronectin-binding protein	100% (12)	100% (12)	69.2% (9)	70.0% (7)	**0.02**
ebps	Cell surface elastin-binding protein	100% (12)	100% (12)	100% (13)	100% (10)	-
eno	Enolase	100% (12)	100% (12)	100% (13)	100% (10)	-
fib	Fibrinogen-binding protein	100% (12)	100% (12)	100% (13)	100% (10)	-
fnbA	Fibronectin-binding protein A	100% (12)	100% (12)	100% (13)	100% (10)	-
fnbB	Fibronectin-binding protein B	83.3% (10)	83.3% (10)	69.2% (9)	100% (10)	0.32
map	Major histocompatibility complex class II analog protein	100% (12)	91.7% (11)	100% (13)	100% (10)	0.72
sasG	*S. aureus* surface protein G	33.3% (4)	58.3% (7)	69.2% (9)	70.0% (7)	0.26
sdrC	Serine-aspartate repeat protein C	100% (12)	100% (12)	100% (13)	100% (10)	-
sdrD	Serine-aspartate repeat protein D	91.7% (11)	91.7% (11)	92.3% (12)	100% (10)	1
vwb	Van Willebrand factor binding protein	100% (12)	100% (12)	100% (13)	100% (10)	-
		**Miscellaneous**				
edinA, edinC	Epidermal cell differentiation inhibitor A+C	0	0	0	0	-
edinB	Epidermal cell differentiation inhibitor B	8.3% (1)	0	7.7% (1)	0	1
*setC**	Staphylococcal exotoxin-like protein	66.7% (8)[g]	91.7% (11)	100% (13)[g]	100% (10)	0.08
ssl6	Staphylococcal superantigen-like protein 6	8.3% (1)[h]	58.3% (7)[h]	38.5% (5)	30.0% (3)	0.08
ssl8	Staphylococcal superantigen-like protein 8	41.7% (5)[i]	91.7% (11)[i]	61.5% (8)	40.0% (4)[i]	**0.034**
ssl11	Staphylococcal superantigen-like protein 11	75.0% (9)	100% (12)[j]	53.8% (7)[j]	40.0% (4)[j]	**0.007**

a: *p* values were calculated with fishers exact test; b: significant difference between nasal colonization and prosthesis infection (p = 0.037), and between prosthesis infection and hematogenous OM (*p* = 0.015); c: significant difference between nasal colonization and prosthesis infection (*p* = 0.027) and prosthesis infection and sepsis (*p* = 0.02); d: significant difference between nasal colonization and prosthesis infection (p = 0.027) and prosthesis infection and sepsis (*p* = 0.02); e: significant difference between prosthesis infection and sepsis (*p* = 0.011); f: significant difference between nasal colonization and prosthesis infection (*p* = 0.047); g: significant difference between prosthesis infection and hematogenous OM (*p* = 0.039); h: significant difference between nasal colonization and prosthesis infection (*p* = 0.027); i: significant difference between nasal colonization and prosthesis infection (*p* = 0.027) and prosthesis infection and sepsis (*p* = 0.02); j: significant difference between prosthesis infection and hematogenous OM (*p* = 0.015) and prosthesis infection and sepsis (*p* = 0.03)

Table S5. Main characteristics of selected strains for long-term cell culture and the mouse sepsis model.

	**N15**	**Chwa42**	**Edbr39**	**N9**	**M1**	**Udmi62**	**D2**
**Group**	**Nasal colonization**	**Hematogenous osteomyelitis**	**Hematogenous osteomyelitis**	**Nasal colonization**	**Prosthesis infection**	**Sepsis**	**Hematogenous osteomyelitis**
**Hemolysis [OD 570nm]**	low	low	low	high	high	high	high
**Invasion [%]**	high	high	high	low	low	low	low
**Cytotoxicity [%]**	low	low	low	high	high	high	high
**cp *hla*/cp *gyrB***	low	low	low	high	high	high	high
**cp *psmα*/cp *gyrB***	low	low	low	high	high	high	high
**cp *agrA*/cp *gyrB***	low	low	low	high	high	high	high
**cp *RNAIII*/cp *gyrB***	low	low	low	high	high	high	high
**Range**	**Hemolysis**	**Invasion [%]**	**Cytotoxicity [%]**	**cp *hla*/cp *gyrB***	**cp *psmα*/cp *gyrB***	**cp *agrA*/cp *gyrB***	**cp *RNAIII*/cp *gyrB***
**low**	0–0.13	2.4–40	7.1–40	0–7	0–250	0.01–2.3	0.1–8
**high**	> 0.13	> 40	> 40	> 7	> 250	> 2.3	> 8

Table S6. Primers used in this study.

**Gene Name**	**Primer Dscription**	**Primer Squence**	**Product Length**
agrA	Forward	5′-AACTGCACATACACGCTTACA-3′	145 nt
	Reverse	5′-GGCAATGAGTCTGTGAGATTT-3′	
hla	Forward	5′-CAACTGATAAAAAAGTAGGCTGGAAAGTGAT-3′	201 nt
	Reverse	5′-CTGGTGAAAACCCTGAAGATAATAGAG-3′	
psmα	Forward	5′-GCCATTCACATGGAATTCGT-3′	151 nt
	Reverse	5′-CAATAGCCATCGTTTTGTCCT-3′	
rnaIII	Forward	5′-TTCACTGTGTCGATAATCCA-3′	70 nt
	Reverse	5′-TGATTTCAATGGCACAAGAT-3′	
gyrB(*)	Forward	5′-AATTGAAGCAGGCTATGTGT-3′	138 nt
	Reverse	5′-ATAGACCATTTTGGTGTTGG-3′	

* Housekeeping gene
